# Are Frontal Cognitive and Atrophy Patterns Different in PSP and bvFTD? A Comparative Neuropsychological and VBM Study

**DOI:** 10.1371/journal.pone.0080353

**Published:** 2013-11-20

**Authors:** Julien Lagarde, Romain Valabrègue, Jean-Christophe Corvol, Fanny Pineau, Isabelle Le Ber, Marie Vidailhet, Bruno Dubois, Richard Levy

**Affiliations:** 1 Department of Neurology, AP-HP, Groupe Hospitalier Pitié-Salpêtrière, Paris, France; 2 Department of Neurology, AP-HP, Hôpital Saint-Antoine, Paris, France; 3 Centre de NeuroImagerie de Recherche (CENIR), Groupe Hospitalier Pitié-Salpêtrière, Paris, France; 4 INSERM, UMR-975, CNRS, UMR-7225, Paris, France; 5 Université Pierre et Marie Curie- Paris 6, Centre de Recherche de l'Institut du Cerveau et de la Moelle épinière (ICM), UMR-S975, Paris, France; 6 INSERM, Centre d'Investigation Clinique, CIC-9503, Groupe Hospitalier Pitié-Salpêtrière, Paris, France; 7 National reference center on rare dementias, AP-HP, Groupe Hospitalier Pitié-Salpêtrière, Paris, France; Centre Hospitalier Universitaire Vaudois Lausanne - CHUV, UNIL, Switzerland

## Abstract

Progressive supranuclear palsy (PSP) and frontotemporal lobar degeneration (FTD) are two clinicohistological entities that share a severe prefrontal syndrome. To what extent do the cognitive syndrome and the location of the underlying brain atrophy unify or segregate these entities? Here, we examined the clinical and radiological patterns of frontal involvement and the neural bases of the cognitive dysfunctions observed in the Richardson form of PSP and the behavioral variant of FTD (bvFTD). The cognitive profile and grey and white matter volume of PSP (n = 19) and bvFTD (n = 16) patients and control participants (n = 18) were compared using a standard battery of neuropsychological tests and voxel-based morphometry (VBM), respectively. Analyses of correlations between neuropsychological and morphometric data were additionally performed. The severity and qualitative pattern of cognitive dysfunction was globally similar between the two patient groups. Grey matter volume was decreased in widespread frontal areas and in the temporal uncus in bvFTD, while it was decreased in the frontal and temporal lobes as well as in the thalamus in PSP. We also found an unexpected involvement of the frontal rectal gyrus in PSP patients compared to controls. Correlation analyses yielded different results in the two groups, with no area showing significant correlations in PSP patients, while several frontal and some temporal areas did so in bvFTD patients. In spite of minor neuropsychological and morphological differences, this study shows that the patterns of cognitive dysfunction and atrophy are very similar in PSP and bvFTD. However, executive dysfunction in these diseases may stem from partially divergent cortical and subcortical neural circuits.

## Introduction

Progressive supranuclear palsy (PSP) and frontotemporal lobar degeneration (FTD) are usually considered two distinct clinicohistological entities. Indeed, on the basis of the clinical syndromes, the topography of brain lesions and their histological/biological characteristics, many features differentiate them: the most frequent clinical form of FTD (called the “behavioral variant”) is a behavioral dementia of the frontal type, while the most typical clinical form of PSP (called the Richardson form) is characterized by axial parkinsonism and supranuclear oculomotor palsy. The topography of neurodegeneration is mostly cortical in FTD, affecting the ventromedial prefrontal cortex (PFC) and to some extent the temporal lobes and dorsal PFC [1], while it is mostly subcortical in PSP, affecting the brainstem, cerebellum and basal ganglia [2]. In terms of the underlying proteinopathies, tau pathology is only found in 20–30% of FTD cases and affects the A, C and E tau isoforms, while PSP is consistently associated with a tauopathy affecting the B, D and F tau isoforms [3].

Despite these important differences, from a clinical standpoint, it is important to note that PSP and FTD share a prominent frontal cognitive syndrome, which is consistently seen in the “classic” Richardson form of PSP. It is present in more than half the patients from the first year of the clinical disease [4], and consists mostly of a dysexecutive syndrome with “cognitive inertia”, i.e. an increased latency of responses, the impairment of information retrieval and reasoning and a lack of mental flexibility [5], [6]. This cognitive syndrome is associated with severe frontal behavioral signs such as apathy/abulia/apragmatism, an environmental dependency syndrome and, more rarely than in bvFTD, behavioral disinhibition [7]–[9]. This frontal cognitive syndrome is sometimes so marked that it has served as the prototypical description of the so-called “subcortical dementia” [10]. However, it is also strongly correlated with frontal hypometabolism [11], more pronounced in the lateral superior and medial PFC [12]. Even though it has been shown to be related to frontal deafferentation due to subcortical lesions (mostly in the basal ganglia) [7], it is now well established that direct cortical lesions involving the frontal cortex do exist [13], and could be implicated in the frontal signs observed in PSP. Conversely, the widespread disruption of subcortical structures has been shown in FTD and could account for some of the observed cognitive or behavioral features of the disease [14], [15]. In addition, frontal dysfunction may appear early in the course of PSP and may present with only one other clinical feature in 20% of patients [16], [17]. Consequently, it is sometimes difficult, at least in the early stages, to clinically distinguish these atypical forms of PSP from the behavioral variant of FTD (bvFTD) [18].

Taken as a whole, these findings suggest that, it would be of interest to compare and clarify the clinical and anatomical features characterizing the frontal cognitive syndrome in PSP and bvFTD. The comparison of these two diseases may provide insights into cognitive changes due to convergent or divergent effects on frontal-lobe-mediated functions. Accordingly, this study addresses a question that has never been considered before: Are the patterns of the frontal cognitive syndrome reported in PSP and bvFTD patients alike or different from the neuropsychological and anatomical perspectives?

## Methods

### General design of the study

The following parameters were compared between PSP and bvFTD patients: 1) the pattern of cognitive dysfunction, using a battery of classic neuropsychological tests to assess rule-finding, set-shifting, verbal concept formation, verbal and motor initiation, and cognitive inhibition, and 2) the level of grey and white matter atrophy, with particular emphasis on frontal regions, using voxel-based morphometry (VBM). Furthermore, we performed a clinicoradiological correlation analysis between grey and white matter volumes and scores obtained on the main components of the executive function tests mentioned above.

### Participants

Fifty-three subjects were prospectively enrolled in this study: 19 patients with PSP, 16 with bvFTD and 18 healthy controls. PSP and bvFTD patients were recruited in the movement disorders unit and the reference center for rare dementias of the Salpêtrière Hospital (Paris, France), respectively. All PSP patients met the NINDS-Society for Progressive Supranuclear Palsy criteria for probable PSP [19], which include a gradually progressing disorder with an onset at or after the age of 40 and vertical supranuclear gaze palsy and prominent postural instability within the first year of disease onset. All bvFTD patients met the consensus criteria for probable frontotemporal dementia [20], [21], presenting with a history of progressive and disabling development of at least three of the six discriminating clinical features associated with characteristic findings on neuroimaging. All patients had a Mini-Mental State Examination (MMSE) score of ≥20/30. Exclusion criteria were previous neurological and psychiatric diseases and the absence of informed consent. The groups were matched for age at the time of the study and educational level, as well as for disease duration in the patient groups. Healthy controls had no history of neurological or psychiatric disorders, no memory or cognitive disorders and no history of psychotropic drug use.

The ethics committee of the Salpêtrière Hospital approved the study. Participants provided written informed consent.

### Neuropsychological evaluation

All subjects underwent a neuropsychological examination. Global intellectual performance was assessed using the Mini Mental State Examination (MMSE) and the Mattis Dementia Rating Scale (MDRS), with the latter providing more emphasis on executive function testing. Frontal functions were evaluated with the Frontal Assessment Battery (FAB), which provides a global bedside evaluation of frontal lobe functions including conceptualization (similarities), mental flexibility (lexical fluency), motor programming (Luria's motor series), sensitivity to interference (conflicting instructions), inhibitory control (go-no go task), and environmental autonomy (grasping behavior) [22], the similarities subtest of the Wechsler Adult Intelligence Scale (WAIS), which tests the ability to find abstract analogies, the modified Wisconsin Card Sorting Test (WCST), which tests abstract rule-finding and set-shifting, letter and category fluency and the Stroop interference task, which evaluates the ability to inhibit cognitive interference. As the three tests assessing concept formation, the WAIS, the FAB, and the MDRS, were redundant, being based on similarities in all cases, we proposed a verbal concept score composed of the sum of the scores obtained on the concept subscale of these tests.

All statistical analyses were performed with Statistica 6 software (StatSoft, Tulsa, OK, USA), using non-parametric tests. We compared demographic and neuropsychological variables between our three groups using a Kruskall-Wallis ANOVA. A Bonferroni correction for five tests (p<0.01) was applied to demographic variables and for eleven tests (p<0.004) to neuropsychological tests.

### Morphological examination

All images were acquired with a 3T MRI scanner on the same day as the neuropsychological examination. Three patients (2 with bvFTD and 1 with PSP) did not undergo MRI. High-resolution three-dimensional T1-weighted MP-RAGE images were acquired using the following parameters: TR (repetition time) of 2.200 ms, TE (echo time) of 2.940 ms, slice thickness of 1 mm, and FOV (field of view) of 256 mm. We also obtained T2-FLAIR images in order to rule out any unnoticed lesions, especially vascular ones. Brain volumes were pre-processed using the VBM8 toolbox on SPM8 software (http://www.fil.ion.ucl.ac.uk/spm). They were segmented into grey matter, white matter and CSF by using a partial volume estimation that evaluated the amount of each pure tissue type present in every voxel, and normalized using high-dimensional spatial normalization to a template derived from 550 healthy control subjects in the IXI-database (http://www.brain-development.org) in MNI space. The images were also modulated, and we chose the option “non-linear only”, in which voxel values are multiplied by non-linear components, which allows the absolute amount of tissue corrected for individual brain sizes to be considered, without entering the total intracranial volume as a covariate in the statistical model. Lastly, the grey matter volume was smoothed with an 8 mm full-width-at-half-maximum Gaussian kernel to minimize individual gyral variations. SPM8 was used for all statistical analyses. Thus, we used voxel-based morphometry (VBM) to compare grey and white matter volumes in the three groups using a full factorial design, with age and sex as nuisance variables [23]. We studied the following contrasts: controls >FTD, controls >PSP, PSP>FTD and FTD>PSP. We also correlated grey and white matter volumes in the bvFTD and PSP groups separately with our neuropsychological scores, using a multiple regression design, with age, sex and MMSE score as nuisance variables and separate design matrices for each executive test. We reported two statistical thresholds: *p*<0.05 with family-wise error (FWE) correction for multiple comparisons at the whole-brain level, and an exploratory threshold of *p*<0.001, uncorrected and taken at a minimal cluster size of 100 voxels, used for the direct comparison of the two patient groups and in correlation analyses.

## Results

### Behavioral data

There was no significant difference between the three groups regarding age at examination, sex or education, nor was there any difference in disease duration or MMSE score between bvFTD and PSP patients (table 1). The proportion of patients with executive impairment (defined by an FAB score of ≤12) was also comparable between the patient groups: 15 out of 19 PSP patients and 10 out of 16 bvFTD patients.

**Table 1:** Comparison of the main demographic parameters between the three groups of subjects.

	FTD	PSP	Controls	*p*
Age at examination (years)	69.25±10	65.9±6.5	67.8±5.2	0.51
Sex (M/F)	9/7	7/12	7/11	0.5
Education (years)	11.75±3.5	11.7±3.9	11.55±2.7	0.51
Disease duration (years) (range)	5.3±3.6 (2–14)	4.5±1.8 (2–9)		1
MMSE (range)	25.1±3.2 (20–30)	25.5±2.7 (20–29)	29.1±0.7 (28–30)	<0.01*

Means ± standard deviations.

* Significant difference using Bonferroni correction for five tests (*p*<0.01). Abbreviations: FTD: frontotemporal dementia; PSP: progressive supranuclear palsy; M: male; F: female; MMSE: Mini-Mental State Examination.

There was no significant difference in any neuropsychological scores between the PSP and the bvFTD group. The interference T-score in the Stroop test tended to be higher in PSP than in bvFTD patients, without reaching statistical significance after correction for multiple comparisons (table 2). When compared to the control group, performance on all the neuropsychological tests was significantly altered in the two groups of patients, except the interference T-score in the Stroop test, which was not significantly different between either group of patients and the control group, even if the mean value in PSP patients was much closer to that of controls than in FTD patients. Furthermore, the verbal concept score was not significantly different between PSP and control groups, although performances tended to be poorer in PSP patients. Memory scores were also altered, especially in bvFTD patients, without reaching statistical significance (table 2).

**Table 2:** Comparison of the main neuropsychological variables between the three groups of subjects.

	FTD	PSP	Controls	*P* global (KW)	FTD vs. PSP	FTD vs. C	PSP vs. C
Verbal concept score	48.5±9.7	52.2±7.9	59.4±5.3	<0.004* (17.6)	0.81	0.002*	0.04
Stroop interference T-score	44.6±6.8	52.6±7.7	50.6±7	0.03 (7.1)	0.02	0.11	1
WCST category number (/6)	3.5±2.1	4.4±1.2	5.8±0.5	<0.004* (19.6)	1	<0.004*	0.004*
WCST perseverance errors	9.3±6.7	6.5±5	1.7±1.4	<0.004* (24.4)	0.99	<0.004*	<0.004*
WCST total Errors	18.6±9.3	14.8±8.1	4.4±3.6	<0.004* (18.6)	1	<0.004*	<0.004*
MDRS attention (/37)	35.9±1	35.1±2.1	36.9±0.3	<0.004* (20.3)	1	0.009	<0.004*
MDRS initiation (/37)	27.5±4.3	29.8±5.7	36.8±0.7	0.00001* (31.8)	1	0.000005*	0.0001*
MDRS memory (/25)	20.3±3.8	22.6±2.5	24.6±0.8	<0.004* (15.45)	0.24	<0.004*	0.026
MDRS total (/144)	124.7±9.3	128.3±10.1	141.8±2	<0.004* (35.4)	1	<0.004*	<0.004*
FAB (/18)	11.7±3.1	11.3±2	17.4±0.6	<0.004* (28.35)	1	<0.004*	<0.004*
Category fluency	18.2±8.1	16.8±7.8	32.6±8.7	<0.004* (17.4)	1	<0.004*	<0.004*
Letter fluency	9.7±6.2	11±6.6	20.1±8.6	0.001* (13.7)	1	0.0008*	0.004

Means ± standard deviations.

* Significant difference using Bonferroni correction for thirteen tests (*p*<0.003). Abbreviations: FTD: frontotemporal dementia; PSP: progressive supranuclear palsy; C: controls; KW: Kruskall-Wallis test; MDRS: Mattis Dementia Rating Scale; FAB: Frontal Assessment Battery; WCST: Wisconsin Card Sorting Test.

### Morphological data

#### Comparison between bvFTD and PSP patients

In bvFTD patients compared to control participants, grey matter volume was significantly decreased in the left frontal rectal gyrus, in the right temporal uncus, in the right medial frontal gyrus, in the right insula, in the right superior and middle frontal gyri, in the mid-cingulate cortex and in the left middle temporal gyrus.

PSP patients, when compared to control participants, showed significantly lower grey matter volume in the left inferior temporal gyrus, the right precentral gyrus, the right rectal gyrus, the right middle temporal gyrus and the anterior nucleus of the right thalamus.

**Figure 1:**
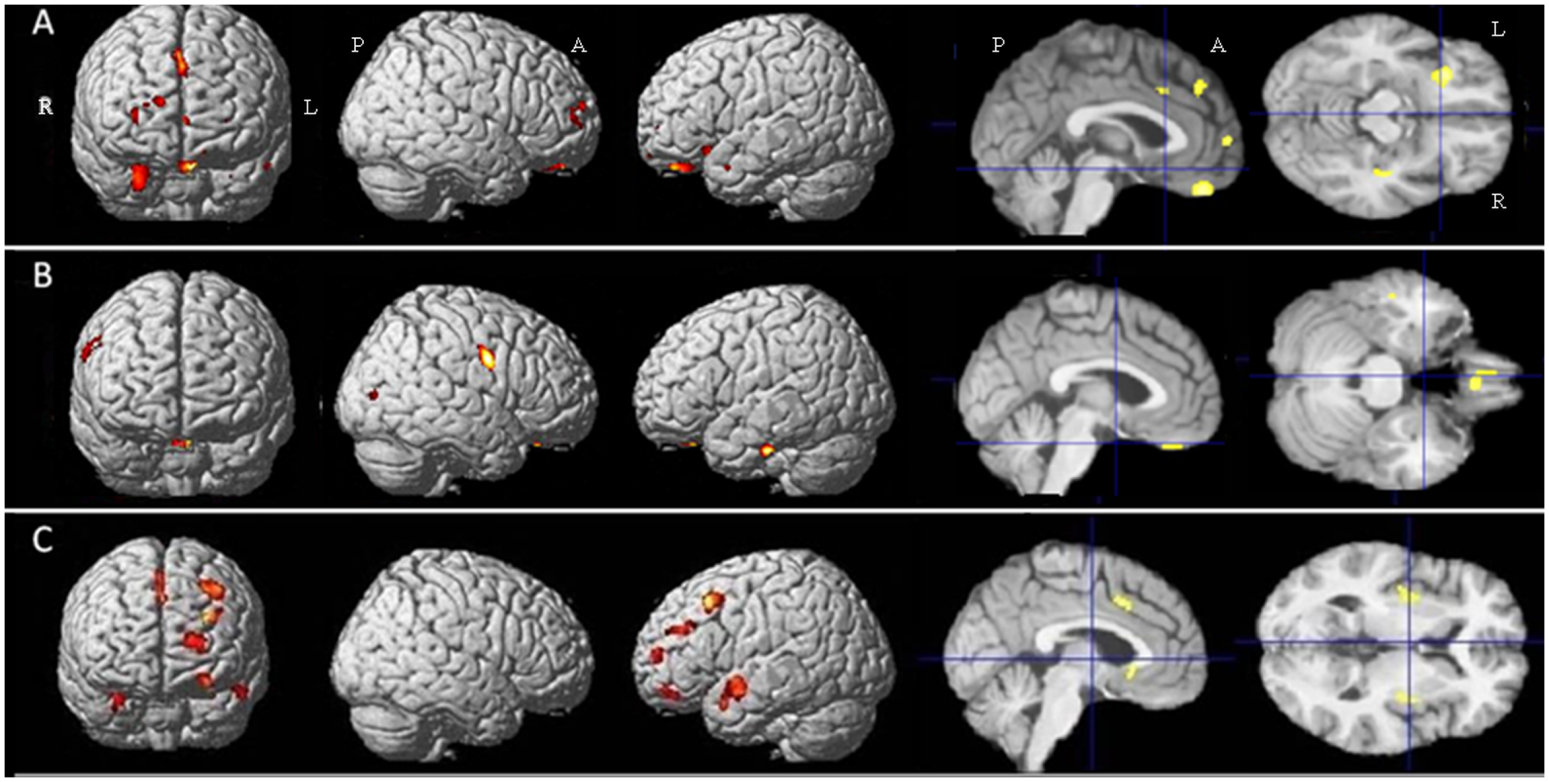
Results of the voxel-based morphometric (VBM) analysis: comparison of grey matter volume between the groups of subjects. A- Zones of decreased grey matter volume in bvFTD patients compared to controls (p<0.05 after FWE correction for multiple comparisons). B- Zones of decreased grey matter volume in PSP patients compared to controls (p<0.05 after FWE correction for multiple comparisons). C- Zones of decreased grey matter volume in bvFTD patients compared to PSP patients (k = 100 voxels, p<0.001). Abbreviations: R: right, L: left, A: anterior, P: posterior, bvFTD: behavioral variant of frontotemporal dementia, PSP: progressive supranuclear palsy.

A direct comparison between the two patient groups did not reveal any significant differences. A more permissive threshold (p<0.001, uncorrected) revealed lower grey matter volume in bvFTD patients than in PSP patients in sparse areas located in the left inferior, middle and superior frontal gyri, the right superior frontal gyrus, the temporal lobes bilaterally (especially the right hippocampus), the left anterior cingulate gyrus, mostly in its subgenual part, as well as near the mid-cingulate cortex and in the right and left putamen. The reverse contrast did not reveal any significant differences (table 3, figure 1).

**Table 3:** Detailed results of the comparative analysis of grey matter volume.

	Localization (BA)	MNI coordinates	Number of voxels	T score
Controls >FTD	Left rectal gyrus (11)	−4 40 −27	339	6.96
	Right uncus (28)	28 8 −26	304	6.64
	Right medial frontal gyrus (8)	4 32 45	300	6.35
	Right insula (13)	36 −9 −18	85	5.46
	Right superior frontal gyrus	15 60 16	60	5.77
	Left middle temporal gyrus (21)	−54 8 −24	16	5.57
	Left cingulate gyrus (32)	−2 17 34	22	5.54
	Right middle frontal gyrus	32 54 9	43	5.53
Controls >PSP	Left inferior temporal gyrus (20)	−51 −19 −30	183	6.9
	Right precentral gyrus	54 −3 31	342	6.57
	Right rectal gyrus (11)	3 28 −29	129	6.1
	Right middle temporal gyrus (19)	50 −81 7	26	5.02
	Right thalamus (ventral anterior nucleus)	9 −12 18	1	5.28
PSP>FTD	Right hippocampus	42 −18 −17	150	4.79
	Left rectal gyrus	−15 24 −14	475	4.63
	Left anterior cingulate (24)	−4 21 −3	475	4.1
	Left middle frontal gyrus (10)	−30 41 28	256	4.32
	Left middle frontal gyrus (8)	−36 20 46	453	4.29
	Right superior frontal gyrus (8)	3 27 58	257	4.19
	Left middle temporal gyrus	−44 −3 −18	827	4.18
	Anterior cingulate (24)	2 11 39	105	4.13
	Right superior temporal gyrus (38)	32 8 −24	175	4
	Right putamen	34 −6 3	195	3.92
	Left superior frontal gyrus (10)	−18 53 13	258	3.9
	Left putamen	−32 −6 1	217	3.8
	Left middle frontal gyrus (11)	−26 51 −12	127	3.74

Areas where grey matter volume is significantly decreased in each patient group compared to controls (p<0.05 after FWE correction for multiple comparisons) and in bvFTD compared to PSP (k = 100 voxels, p<0.001 uncorrected). Abbreviations: BA: Brodmann area; MNI: Montreal Neurological Institute; FTD: frontotemporal dementia; PSP: progressive supranuclear palsy.

**Figure 2:**
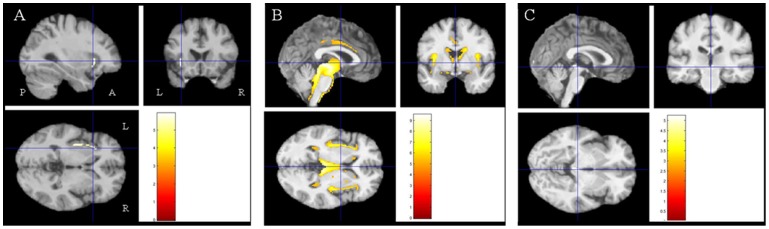
Results of the voxel-based morphometric (VBM) analysis: comparison of white matter volume between the groups of subjects. A- Zones of decreased white matter volume in bvFTD patients compared to controls (p<0.05 after FWE correction for multiple comparisons). B- Zones of decreased white matter volume in PSP patients compared to controls (p<0.05 after FWE correction for multiple comparisons). C- Zones of decreased white matter volume in PSP patients compared to bvFTD patients (p<0.05 after FWE correction for multiple comparisons). Abbreviations: R: right, L: left, A: anterior, P: posterior, bvFTD: behavioral variant of frontotemporal dementia, PSP: progressive supranuclear palsy.

Comparisons between patient groups and controls showed a marked decrease in white matter volume in widespread subcortical regions in PSP patients and in the left insula in bvFTD patients. The direct comparison between our two groups of patients showed lower white matter volume in the cerebral peduncles for PSP patients. There was no significant decrease in white matter volume in bvFTD patients when compared with PSP patients (table 4, figure 2).

**Table 4:** Detailed results of the comparative analysis of white matter volume.

	Localization	MNI coordinates	Number of voxels	T score
Controls >FTD	Left insula	−32 18 −5	215	5.93
Controls >PSP	Midbrain	−2 −21 −5	12829	9.53
	Right claustrum	36 −13 0	1936	7.21
	Right middle frontal gyrus	24 18 43	117	6.59
	Right parietal lobe	34 −34 43	158	6.51
	Right precentral gyrus	44 −10 31	150	6.11
	Right cingulate gyrus	9 8 42	195	5.93
	Left cingulate gyrus	−6 15 31	478	5.91
FTD>PSP	Midbrain	2 −28 −6	57	5.23

Areas where white matter volume is significantly decreased in each patient group compared to controls and in PSP compared to bvFTD (p<0.05 after FWE correction for multiple comparisons). Abbreviations: MNI: Montreal Neurological Institute; FTD: frontotemporal dementia; PSP: progressive supranuclear palsy.

#### Correlations between neuropsychological and morphological data in patients

**Figure 3:**
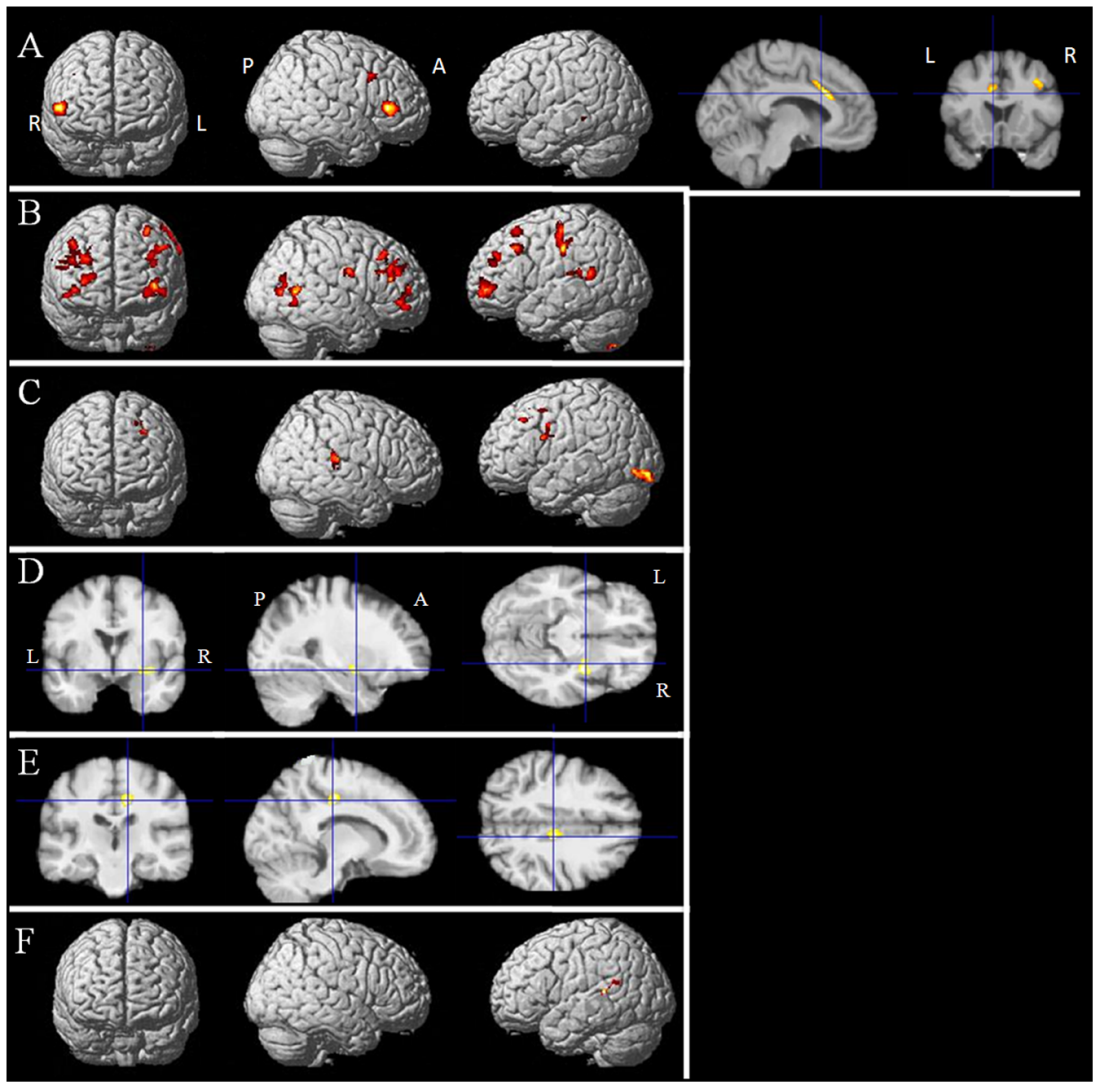
Results of the voxel-based morphometric (VBM) analysis: correlations between grey matter volume and neuropsychological variables in bvFTD patients. A- Positive correlation between grey matter volume and the interference T-score in the Stroop test (k = 100 voxels, p<0.001). B- Positive correlation between grey matter volume and the number of categories found in the WCST (k = 100 voxels, p<0.001). C- Positive correlation between grey matter volume and letter fluency (k = 100 voxels, p<0.001). D- Positive correlation between grey matter volume and the MDRS initiation score (k = 100 voxels, p<0.001). E- Positive correlation between grey matter volume and the MDRS attention score (k = 100 voxels, p<0.001). F- Positive correlation between grey matter volume and the concept score (k = 100 voxels, p<0.001). Abbreviations: R: right, L: left, A: anterior, P: posterior, WCST: Wisconsin Card Sorting Test, MDRS: Mattis Dementia Rating Scale.

**Figure 4:**
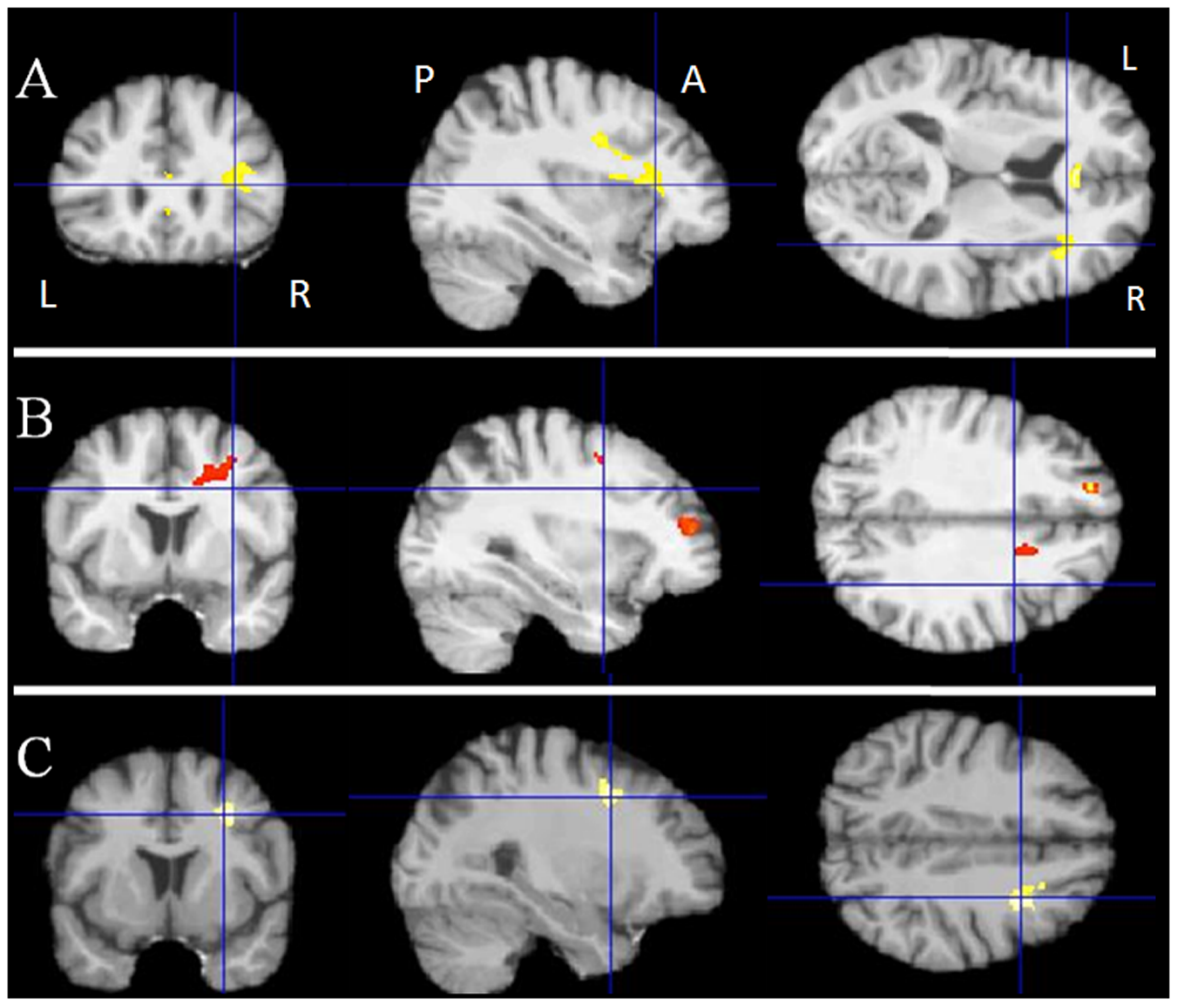
Results of the voxel-based morphometric (VBM) analysis: correlations between white matter volume and neuropsychological variables in bvFTD patients. A- Positive correlation between white matter volume and the interference T-score in the Stroop test (k = 100 voxels, p<0.001). B- Positive correlation between white matter volume and the number of categories found in the WCST (k = 100 voxels, p<0.001). C- Positive correlation between white matter volume and the MDRS attention score (k = 100 voxels, p<0.001). Abbreviations: R: right, L: left, A: anterior, P: posterior, WCST: Wisconsin Card Sorting Test, MDRS: Mattis Dementia Rating Scale.

The differences found between the cognitive profiles and grey and white matter volumes of the two subgroups of patients led us to perform separate correlation analyses in order to study the neural bases of the cognitive functions that were altered in these pathological conditions. Significant positive correlations were noted between grey matter volume and neuropsychological scores (i.e., lower grey matter volume with lower scores) in bvFTD patients as follows: 1) the interference T-score of the Stroop test with the left mid-cingulate cortex, the right inferior and middle frontal gyri and the left superior temporal gyrus (figure 3A); 2) the number of categories found in the WCST and the left and right middle frontal gyri, the right middle temporal gyrus and the left superior temporal gyrus (figure 3B); 3) letter fluency and the left inferior occipital gyrus, the right posterior cingulate, the left inferior and middle frontal gyri, the left insula and the right superior temporal gyrus (figure 3C); 4) the initiation score of the MDRS and the right inferior frontal gyrus (figure 3D); 5) the attention score of the MDRS and the right cingulate gyrus (figure 3E); 6) the concept score and the left superior temporal gyrus (figure 3F, table 5). No significant correlation was found for the other neuropsychological variables.

**Table 5:** Detailed results of the correlation analysis of grey matter volume in bvFTD patients.

	Localization (BA)	MNI coordinates	Number of voxels	T score
Stroop Interference T-score	Left anterior cingulate (24)	−10 20 27	405	7.50
	Right inferior frontal gyrus	52 30 6	633	7.02
	Right middle frontal gyrus	34 14 39	149	6.01
	Left superior temporal gyrus	−45 −39 0	136	5.24
	Right fusiform gyrus	27 −75 −6	281	6.15
	Left middle frontal gyrus	−42 56 10	1434	5.35
	Right middle frontal gyrus (10)	38 39 24	2414	5.33
	Right middle frontal gyrus	48 20 30	864	5.16
WCST number of categories	Left precentral gyrus	−63 −22 39	679	13.25
	Right middle frontal gyrus	38 40 −2	249	12.04
	Right middle frontal gyrus	44 32 46	220	12
	Right middle temporal gyrus	66 −61 7	316	10.53
	Left superior temporal gyrus (22)	−56 −48 15	191	8.66
	Left middle temporal gyrus (9)	−32 41 30	222	8.06
	Right middle temporal gyrus (39)	52 −72 7	387	7.95
	Left middle frontal gyrus (10)	−38 56 4	603	7.08
	Left middle frontal gyrus (9)	−50 26 40	212	5.78
Letter fluency	Left inferior occipital gyrus	−28 −91 −11	445	7.42
	Right posterior cingulate	4 −51 6	192	7.37
	Left middle frontal gyrus	−28 12 40	470	6.58
	Left inferior frontal gyrus	−39 11 22	264	6.05
	Left insula	−42 −19 0	159	5.82
	Right superior temporal gyrus	51 −30 15	216	5.45
MDRS initiation score	Right inferior frontal gyrus	33 −4 −12	224	6.03
MDRS attention score	Right cingulate gyrus	12 −22 40	214	6.33
Concept score	Left superior temporal gyrus (13)	−45 −46 19	126	8.39

Areas where grey matter volume is positively correlated with the interference T-score in the Stroop test, the number of categories found in the WCST, the MDRS initiation score, the MDRS attention score, and the concept score in bvFTD patients (k = 100 voxels, p<0.001 uncorrected). Abbreviations: BA: Brodmann area; MNI: Montreal Neurological Institute; WCST: Wisconsin Card Sorting Test; MDRS: Mattis Dementia Rating Scale.

A significant positive correlation was noted between letter fluency and grey matter volume in the left and right middle frontal gyri of PSP patients. No significant correlation was found for the other neuropsychological variables.

Significant positive correlations were noted between white matter volume and neuropsychological scores in bvFTD patients as follows: 1) the interference T-score in the Stroop test and the corpus callosum and right frontal lobe white matter (figure 4A); 2) the number of categories found in the WCST and the right middle and medial frontal lobe, left superior frontal lobe and cingulate region (figure 4B); 3) the attention score of the MDRS and the right frontal lobe (figure 4C, table 6). No significant correlation was found for the other neuropsychological variables.

**Table 6:** Detailed results of the correlation analysis of white matter volume in bvFTD patients.

	Localization	MNI coordinates	Number of voxels	T score
Stroop Interference T- score	Corpus callosum	2 32 6	227	6.51
	Right frontal lobe	40 22 7	725	5.41
WCST number of categories	Left superior frontal gyrus	−15 45 33	131	11.19
	Right medial frontal gyrus	10 64 0	200	7.55
	Right middle frontal gyrus	32 46 15	100	6.85
	Right cingulate gyrus	15 9 37	371	5.39
MDRS attention score	Right frontal lobe	32 8 39	295	5.74

Areas where white matter volume is positively correlated with the interference T-score in the Stroop test, the number of categories found in the WCST, and the attention score in the MDRS in bvFTD patients (k = 100 voxels, p<0.001 uncorrected). Abbreviations: MNI: Montreal Neurological Institute; WCST: Wisconsin Card Sorting Test; MDRS: Mattis Dementia Rating Scale.

Correlations between white matter volume and the neuropsychological scores in PSP patients were not significant.

## Discussion

In this study, we compare executive dysfunction and atrophy patterns between PSP and bvFTD using a morphometric method, and perform clinicoradiological correlation analysis at the whole-brain level (i.e. without any prespecified region of interest). To our knowledge, this is the first time that these parameters have been directly compared in these pathological conditions.

Frontal dysexecutive syndrome is an important feature of both bvFTD and PSP patients. Interestingly, our neuropsychological data failed to reveal cognitive profiles distinct to each of the two pathologies. However, the underlying mechanisms could be different, and we cannot rule out the possibility that cognitive slowing leads to impaired performances on executive tasks in PSP. Nevertheless, the interference T-score in the Stroop test tended to be higher in PSP than in bvFTD patients, even if the difference did not persist after correction for multiple comparisons. Further studies are required to determine whether or not this cognitive difference could be helpful in dissociating these two clinical entities, in addition to the presence of a gait disturbance that has been shown to be the most sensitive marker of PSP in a clinicopathological study [24] and to the motor control impairment seen in PSP, which is also of use in discriminating PSP from bvFTD [25]. Another potential discriminating factor is the presence of memory deficits in bvFTD, as suggested in previous studies [14]. The behavioral characteristics of these two entities have not been addressed in the present study but have previously been compared elsewhere [9]: apathy is a frequent feature of both PSP and bvFTD, unlike disinhibition, which is observed more rarely in PSP [26], [27].

The pattern of atrophy seen in the present study is globally in line with previous reports in which PSP and bvFTD have been studied separately. Indeed, frontal atrophy has been associated in other studies with bvFTD, in comparison to healthy controls or patients with Alzheimer's disease [28]. Some authors have reported a left-sided hypometabolism in bvFTD [29]. The most affected regions are the orbitofrontal, ventromedial frontopolar, insular and anterior cingulate cortices [1]. On the other hand, PSP has been associated with atrophy in the thalamus, superior and inferior colliculi, striatum, pons and frontal cortex, especially the supplementary motor areas, left middle frontal gyrus and premotor cortex [30]. However, the direct comparison between PSP and bvFTD performed in the present study provides additional information. First, the pattern of atrophy was globally very similar between PSP and bvFTD. Second, one could nevertheless observe some differences between these two entities in the present study: when compared to controls, atrophy in bvFTD patients was more marked in the rectal gyrus, in medial frontal regions and bilaterally within the temporal lobes, particularly in the hippocampus, as reported in previous studies [14], [31]. Conversely, changes in PSP (as compared to bvFTD) were more marked, as expected, in the cerebral peduncles, affecting white matter volume. Unexpectedly, we found decreased grey matter volume in the rectal gyrus in PSP patients (in comparison with controls). Even though this pattern is better known in bvFTD, it is in line with studies in which behavioral features in PSP patients were associated with volume loss in the frontal lobe [26], especially in the most ventral part of the prefrontal cortex, including the orbitofrontal region [32], which is known to be involved in behavioral control and social cognition [33]. In particular, a recently published study has shown a positive correlation between poorer performance in social cognition tasks and volume loss in the anterior rostral medial prefrontal cortex in PSP patients [34].

Correlations between cortical atrophy or hypoperfusion and global cognitive or behavioral scores have already been studied in these two entities separately, and most often, without distinguishing between different regions of the frontal lobes. Indeed, right frontal lobe hypoperfusion in bvFTD has been associated in previous studies with a loss of insight, environmental dependency and stereotyped behaviors [35]. Along the same line of thought, medial frontal and cingulate hypoperfusion has been linked to inertia, whereas ventromedial dysfunction is related to disinhibition [36]. Frontal lobe atrophy has also been correlated with clinical dementia and with the worsening of executive functions in PSP [37], [38]. In the present work, we separately studied the neural bases of several cognitive functions in these two neurodegenerative diseases, with the aid of our detailed neuropsychological battery. The most interesting result was the significant correlation between interference T-score in the Stroop test, which reflects cognitive inhibition, and grey matter volume in the mid-cingulate cortex and the inferior and middle frontal gyri in bvFTD. This result is in accordance with the putative role of the anterior cingulate in conflict monitoring and processing [39], and of the right inferior frontal gyrus in cognitive inhibition [40]. Furthermore, in bvFTD patients, the number of categories found in the WCST, which reflects rule-finding ability, was associated with bilateral changes in the dorsolateral prefrontal areas. Letter fluency was mainly correlated with the volume of the left frontal regions in bvFTD patients, in accordance with previous reports [41]. The initiation score of the MDRS, which also assesses perseverance, was correlated with changes in the right inferior frontal area, known to be involved in cognitive inhibition as mentioned above [40]. The attention score of the MDRS was correlated with changes in the right cingulate gyrus, pinpointing the important contribution of the atrophy of the mid-cingulate cortex to the cognitive control impairment observed in this group of bvFTD patients, in accordance with the presumed role of this multifunctional region in cognition [42]. Lastly, the concept score was correlated with left superior temporal atrophy.

The lack of significant correlations in PSP suggests that the underlying brain correlates of cognitive dysfunctions might be different than in bvFTD, involving more widespread atrophy of subcortical and cortical grey and white matter. This dissemination of lesions in PSP, combined with the relatively small size of the sample may have prevented us from detecting significantly modified areas in our correlation analyses due to the lack of statistical power.

The present study has other limitations that must be acknowledged. First, the great variability in the scores on cognitive tests together with the limited number of subjects might have affected our ability to detect differences between the patient groups. Second, the number of participants was relatively small, especially in the bvFTD group, even if it was similar or larger than in other VBM studies [30]. The low sample size could have led to a lack of power, preventing us from finding significant correlations between some neuropsychological variables and anatomical changes. Lastly, VBM is not the best method to assess white matter volume. This could account for our inability to find significant correlations, especially in PSP patients, who show a massive decrease in white matter volume that could contribute to the observed cognitive impairment.

## Conclusion

Beyond the apparent homogeneity of the clinical frontal syndrome in bvFTD and PSP, subtle but significant differences indicate that cognitive impairment in the two clinical entities could stem from partially divergent neural circuits, including both cortical and subcortical regions. The similar patterns of cognitive dysfunction and atrophy in PSP and bvFTD should nevertheless draw the attention of clinicians to the difficulty in distinguishing between these two entities on the basis of cognition in atypical cases presenting with an isolated progressive frontal syndrome.
